# Laparoscopic partial liver resection for hepatocellular carcinoma arising from Fontan-associated liver disease: a case report

**DOI:** 10.1186/s40792-021-01198-4

**Published:** 2021-05-10

**Authors:** Miku Iwata, Katsunori Sakamoto, Chihiro Ito, Akimasa Sakamoto, Mio Uraoka, Tomoyuki Nagaoka, Kei Tamura, Naotake Funamizu, Akihiro Takai, Kohei Ogawa, Yasutsugu Takada

**Affiliations:** Department of Hepato-Biliary-Pancreatic and Breast Surgery, Ehime University Graduate School of Medicine, Shitsukawa, Toon, Ehime 791-0295 Japan

**Keywords:** Fontan-associated liver disease, Hepatocellular carcinoma, Laparoscopic surgery

## Abstract

**Background:**

The Fontan procedure (FP) is a palliative surgery for functional single ventricle. The Fontan circulation maintains pulmonary circulation by a high central venous pressure, leading to chronic congestive liver. The number of patients diagnosed with hepatocellular carcinoma (HCC) arising from liver fibrosis and cirrhosis after FP is increasing. Several reports have described surgical treatment for HCC after FP, but few have described laparoscopic surgery.

**Case presentation:**

The patient was a 31-year-old man who had undergone the FP for single right ventricle at 3 years. Several liver masses were detected at 30 years. A liver mass in segment 3 showed increasing size concomitant with increasing alpha-fetoprotein concentration, and a solitary HCC 15 mm in diameter was diagnosed. The tumor was located on the liver surface, abutting the origin of the left hepatic vein. Laparoscopic partial liver resection was performed. The postoperative course was uneventful and the patient was discharged on postoperative day 3. The patient remained disease-free on follow-up after 7 months.

**Conclusions:**

Although we had some concerns, such as difficulty managing general anesthesia and easy venous bleeding due to high central venous pressure, laparoscopic partial liver resection was performed with safe exposure of the left hepatic vein.

## Background

The Fontan procedure (FP) is a palliative surgery for functional single ventricle. The Fontan circulation maintains pulmonary circulation through a high central venous pressure (CVP), leading to chronic congestive liver [1]. Recently, the long-term prognosis after FP has been improving thanks to advances in both procedures and postoperative management [1]. The number of patients diagnosed with late complications of hepatocellular carcinoma (HCC) arising from liver fibrosis and cirrhosis, as so-called Fontan-associated liver disease (FALD), is, therefore, increasing [1]. Although several reports have described liver resection for HCC arising from FALD [1, 2], laparoscopic liver resection has rarely been reported. Laparoscopic liver resection for HCC arising from FALD might tend to be avoided because of the potential for difficulties in both controlling venous bleeding due to the high CVP and anesthetic management [2]. Herein, we report a case of HCC that developed 28 years after FP and was treated with laparoscopic partial liver resection exposing a major hepatic vein.

## Case presentation

**Fig. 1:**
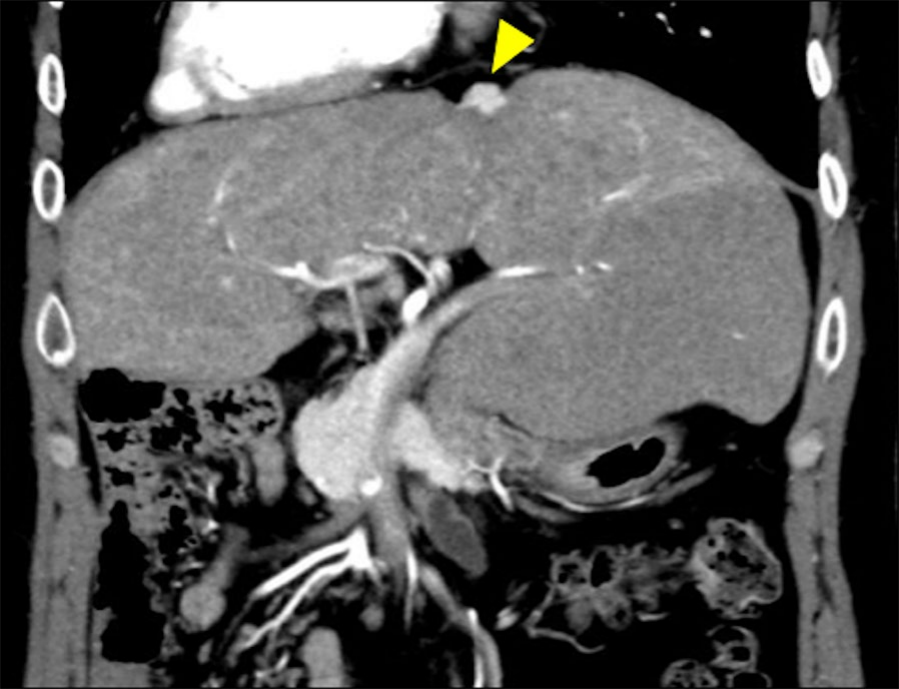
Abdominal dynamic contrast-enhanced CT (arterial phase). Arrowhead shows the tumor protruding from the liver surface

The patient was a 31-year-old man with single right ventricle and congenital asplenia syndrome who had undergone FP at 3 years. Several liver masses were detected at 30 years. One tumor was diagnosed as focal nodular hyperplasia from ultrasound-guided biopsy, and close follow-up was maintained. At 31 years, abdominal dynamic contrast-enhanced computed tomography (CT) revealed another S3 liver mass that had enlarged to 15 mm in diameter. This tumor was diagnosed as HCC based on the appearance of high density from the arterial phase to the portal phase and wash-out in the equilibrium phase (Fig. 1). Hepatic arteriography revealed no other intrahepatic lesions and solitary HCC was thus diagnosed (cT1N0M0, Stage I according to the 8^th^ edition of the classification of the Union for International Cancer Control [3]). The HCC in S3 was located on the liver surface, abutting the origin of the left hepatic vein. Preoperative CT revealed no ascites or collateral circulation. Blood testing showed: aspartate transaminase, 34 U/L; alanine transaminase, 52 U/L; albumin, 4.2 mg/dL; total bilirubin, 1.6 mg/dL; indirect bilirubin, 0.3 mg/dL; prothrombin time-international normalized ratio, 1.01; and platelet count, 18.9 × 10^4^/μL. Alpha-fetoprotein and des-gamma-carboxy prothrombin were elevated to 277.8 ng/mL and 56 mAU/mL, respectively. Type IV collagen 7S was slightly elevated to 8.4 ng/mL, but other markers of liver fibrosis were normal (hyaluronic acid, 29 ng/mL; Mac-2-binding protein glycosylation isomer, 0.51 cut-off index). Negative results were obtained for both hepatitis B virus surface antigen and hepatitis C virus antibody, and the patient had no history of alcohol consumption. The indocyanine green (ICG) retention rate at 15 min was 44%. Ratios of HH 15 (representing blood clearance) and LHL 15 (representing hepatic uptake) on ^99m^Tc-GSA scintigraphy were 0.71 and 0.95, respectively. Child–Pugh classification was A. Echocardiography demonstrated good single right ventricular function and no obstruction in the Fontan circulation. Fractional area change was 42.4%, and common atrioventricular valve regurgitation was mild. Oxygen saturation in room air was 89%.

**Fig. 2:**
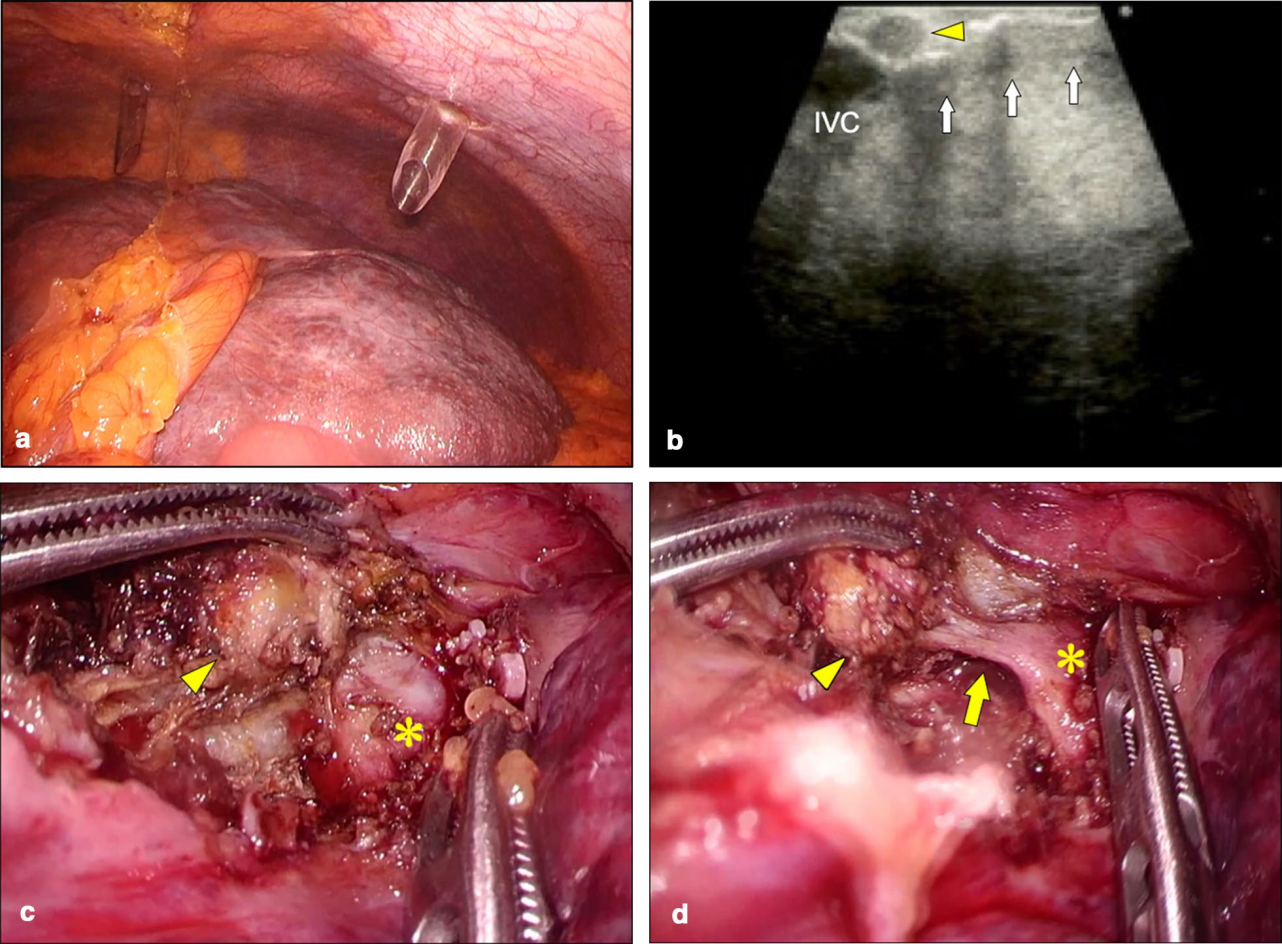
Operative findings. **a** Intraoperative findings of the liver show congestive hepatomegaly and cirrhosis. **b** Intraoperative ultrasonography demonstrates the S3 tumor (yellow arrowhead) abutting the inferior vena cava (IVC) and left hepatic vein (white arrow). **c** Tumor (yellow arrowhead) is abutting the left hepatic vein (*). **d** Tumor (yellow arrowhead) is enucleated with exposure of the left hepatic vein (*). Yellow arrow shows the tributary to the origin of the left hepatic vein from the tumor

**Fig. 3:**
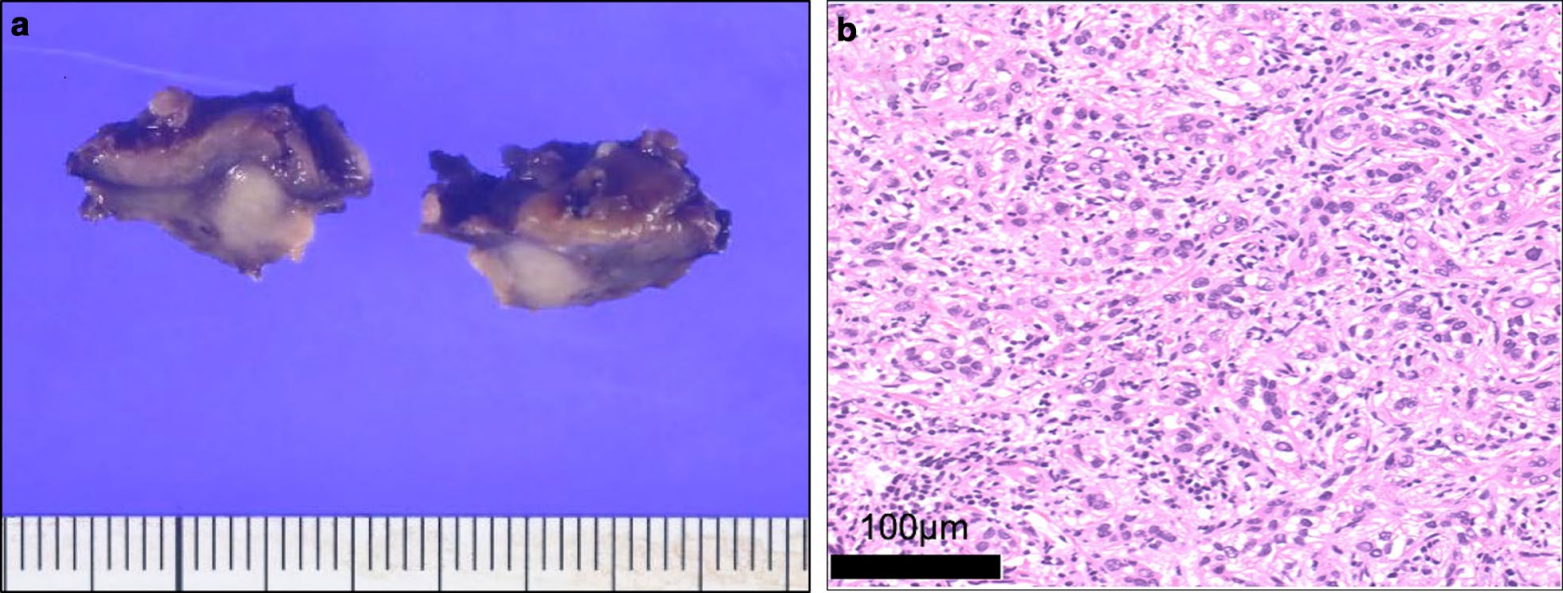
Histopathological examination of the resected specimen. **a** Macroscopic examination shows a white, encapsulated solid tumor, 10 mm in maximum diameter. Surgical margins are negative, but 0 mm. **b** Microscopic examination shows irregular, cord-like pattern cells with eosinophilic cytoplasm and nuclear atypia. The pathological diagnosis is well-to-moderately differentiated hepatocellular carcinoma (hematoxylin and eosin staining; ×10)

We decided to perform laparoscopic partial liver resection after a multidisciplinary discussion with the cardiologist and anesthesiologist. After induction of general anesthesia, a central venous catheter was inserted into the right internal jugular vein for intraoperative monitoring of CVP. A transesophageal echocardiogram was also placed. The patient was placed supine, then four trocars and one tourniquet for the Pringle maneuver were positioned. Pneumoperitoneum was started at a pressure of 8 mmHg and brought up to 10 mmHg to achieve a better surgical field while carefully monitoring vital signs. CVP was elevated from 11 to 14 mmHg after reaching pneumoperitoneum of 10 mmHg and systolic blood pressure also elevated from 80 to 100 mmHg. Macroscopic examination of the liver showed cirrhosis (Fig. 2a). By dissecting the coronary ligament of the liver, the suprahepatic inferior vena cava (IVC) was exposed. Intraoperative ultrasonography identified the S3 tumor abutting the origin of the left hepatic vein (Fig. 2b). The liver parenchyma was transected using cavitron ultrasonic surgical aspirator (Integra Lifesciences Corporation, Plainsboro, NJ, USA), and the tumor was enucleated, exposing the anterior aspect of the left hepatic vein (Fig. 2c). The venous tributary from the tumor was cut at its origin on the left hepatic vein (Fig. 2d). Neither injury nor bleeding occurred. The surgery lasted 117 min, and estimated blood loss was 10 mL. Since no bleeding from hepatic veins occurred, the Pringle maneuver and a change to a reverse Trendelenburg position were not used. Although intermittent multiple premature ventricular contractions occurred intraoperatively, systolic blood pressure remained stable at almost 100 mmHg. After finishing pneumoperitoneum, CVP decreased to 7 mmHg with systolic blood pressure at 100 mmHg. The surgical margin was 0 mm, but negative (Fig. 3a). On histopathological examination, the tumor was diagnosed as moderately to well-differentiated HCC (Fig. 3b), and peritumoral liver tissue showed stage F4 cirrhosis according to the new Inuyama classification [4].

The postoperative course was uneventful and the patient was discharged on postoperative day 3. As of the 7-month follow-up, the patient remained disease-free.

## Discussion

Liver dysfunction arising from FALD causes liver fibrosis, cirrhosis and HCC, even in young patients [5]. FALD results from fibrosis of the sinusoidal and portal canals due to excessive liver congestion caused by the high CVP. The stage of FALD depends on the duration after FP and hepatic venous pressure [6]. FALD can develop to liver cirrhosis as early as 11–15 years after FP, and the cumulative incidences of cirrhosis at 20 and 30 years after FP have been reported as 56.6% and 97.9%, respectively [7]. The incidence of HCC in FALD has been estimated as 1.5–5.0% per year [8]. Our patient presented 28 years after FP with no relevant symptoms or blood biochemistry results, strongly suggesting liver fibrosis or cirrhosis.

ICG has commonly been used to evaluate preoperative liver function in Japan, but accurate evaluation is difficult in the presence of factors, such as portosystemic shunt [9]. However, ^99m^Tc-GSA scintigraphy is unaffected by these pathologies [9]. Even though the present case did not require detailed evaluation of liver function, because the small partial hepatectomy was sufficient for cancer treatment, preoperative evaluation of liver function might be difficult for patients with FALD, as previously reported [10]. Since the ICG retention rate at 15 min in the present study indicated poor liver function, ^99m^Tc-GSA scintigraphy was added for further assessment of preoperative liver function. In the results from ^99m^Tc-GSA scintigraphy, LHL 15 indicated favorable liver function, whereas HH 15 indicated poor liver function. This discrepancy between LHL 15 and HH 15 might be explained by the low H 3, representing radioactivity in the cardiac region of interest at 3 min after injection. In the Fontan circulation, systemic–pulmonary artery shunt may result in a low H 3. In fact, the H 3 value of the present case was relatively lower than that of various other patients in our experience (data not shown). However, since detailed reports of FALD and ^99m^Tc-GSA scintigraphy have not been reported previously, further study is required regarding the assessment of liver function using ^99m^Tc-GSA scintigraphy in cases of FALD. Finally, histopathological examination in the present case demonstrated liver cirrhosis.

Most cases of HCC arising from FALD are reportedly treated non-surgically, with poor liver function reducing the tolerance for surgical resection [11]. However, recent improvements in surgical procedures and perioperative management have enabled safe liver resection not only by laparotomy, but also in laparoscopic surgery [12, 13]. The present case appears to represent the third report of laparoscopic liver resection for HCC arising from FALD [12, 13], but the first case in which the origin of a hepatic vein was exposed. Laparoscopic surgery shows some limitations for patients who have undergone FP. Laparoscopic surgery easily leads to low cardiac output due to the elevations in intrathoracic and intraabdominal pressures under positive-pressure ventilation and pneumoperitoneum [12, 13]. Nonetheless, although CVP was elevated after the initiation of pneumoperitoneum in our case, blood pressure was kept stable. Injection of sufficient fluid (about 500 mL/h during anesthesia) might have helped maintain the stable condition in the present case. However, uncontrollable bleeding from hepatic veins may occur readily if a hepatic vein is injured. Although we were able to successfully perform laparoscopic hepatectomy exposing a hepatic vein, we had to prepare options in case of bleeding from a hepatic vein, such as the reverse Trendelenburg position or Pringle maneuver. As noted in the previous study, the Pringle maneuver does not have adverse effects on the Fontan circulation during laparoscopic hepatectomy [13]. On the other hand, IVC clamping can easily lead to low blood pressure in the Fontan circulation [1]. We did not apply the reverse Trendelenburg position during the operation due to concerns regarding unstable blood pressure with decreases in venous return [13], but a recent study suggested the utility of this position to reduce CVP with stable vital signs [1]. However, since no reports have described laparoscopic liver resection applying a reverse Trendelenburg position, further study is required. In addition, although we did not encounter any elevation of the airway pressure to over 20 cmH_2_O in the present case, strict control of airway pressure may also be required to control bleeding from hepatic veins especially in laparoscopic hepatectomy for patients with FALD [14].

The indications for laparoscopic liver resection among patients with FALD thus have to be determined carefully with close communication between the cardiologist and anesthesiologist. Furthermore, we have to keep in mind the timing of conversion to open surgery when performing laparoscopic surgery, not only because of the risk of intraoperative bleeding, but also because changes in vital signs may easily arise in patients with FALD. Liver resection to treat HCC arising from FALD, therefore, requires stricter criteria than conventional liver resection. Such criteria may include preoperative CVP and wedge pressure of the hepatic veins to predict the likelihood of intraoperative bleeding from hepatic veins. To establish such criteria, we should accumulate data on patients with FALD by creating a large-scale, nationwide database.

In the present case, a hepatic vein had to be exposed to remove the tumor. However, the wall of the hepatic vein showed fibrous thickening rather than fragility, and no bleeding was observed. Liver congestion is known to lead to fibrous thickening of veins, such as the IVC and hepatic veins [15]. The fibrous thickening of the hepatic vein in this case might have been caused by prolonged liver congestion or inflammation due to the tumor, but the precise reasons remain unclear. Further cases need to be accumulated to clarify such anatomical changes.

## Conclusions

We have reported a case of HCC abutting the origin of the left hepatic vein after FP, treated successfully with laparoscopic partial liver resection. We hope that the present case will help in the treatment of patients with HCC arising from FALD.

## Data Availability

The authors declare that all the data in this article are available within the article.

## Abbreviations

FP: Fontan procedure
CVP: Central venous pressure
HCC: Hepatocellular carcinoma
FALD: Fontan-associated liver disease
CT: Computed tomography
ICG: Indocyanine green
IVC: Inferior vena cava
